# Supplementary material for: Dissection of Hyperspectral Reflectance to Estimate Photosynthetic Characteristics in Upland Cotton (Gossypium hirsutum L.) under Different Nitrogen Fertilizer Application Based on Machine Learning Algorithms
Source: Plants (Basel). 2023 Jan 19;12(3):455. doi: 10.3390/plants12030455 (PMC9919998; doi:10.3390/plants12030455)
Supplement: Supplementary file 1 [file plants-12-00455-s001.zip › plants-1918524-Supplementary Figures.pdf]

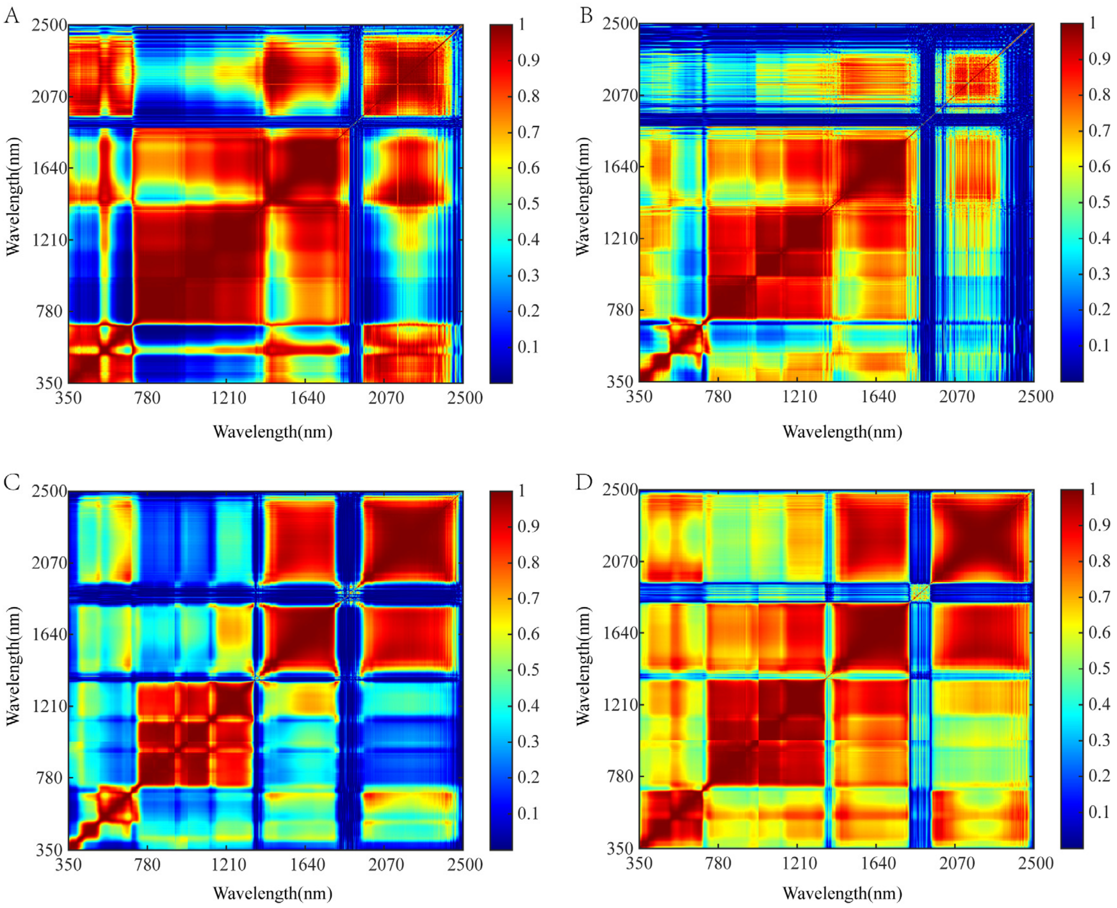

Figure S1. Autocorrelation of spectral reflectance in different bands of cotton canopy at different growth-stages. Note: A is full flowering stage; B is flower and boll stage; C is full boll stage; D is early boll opening stage.

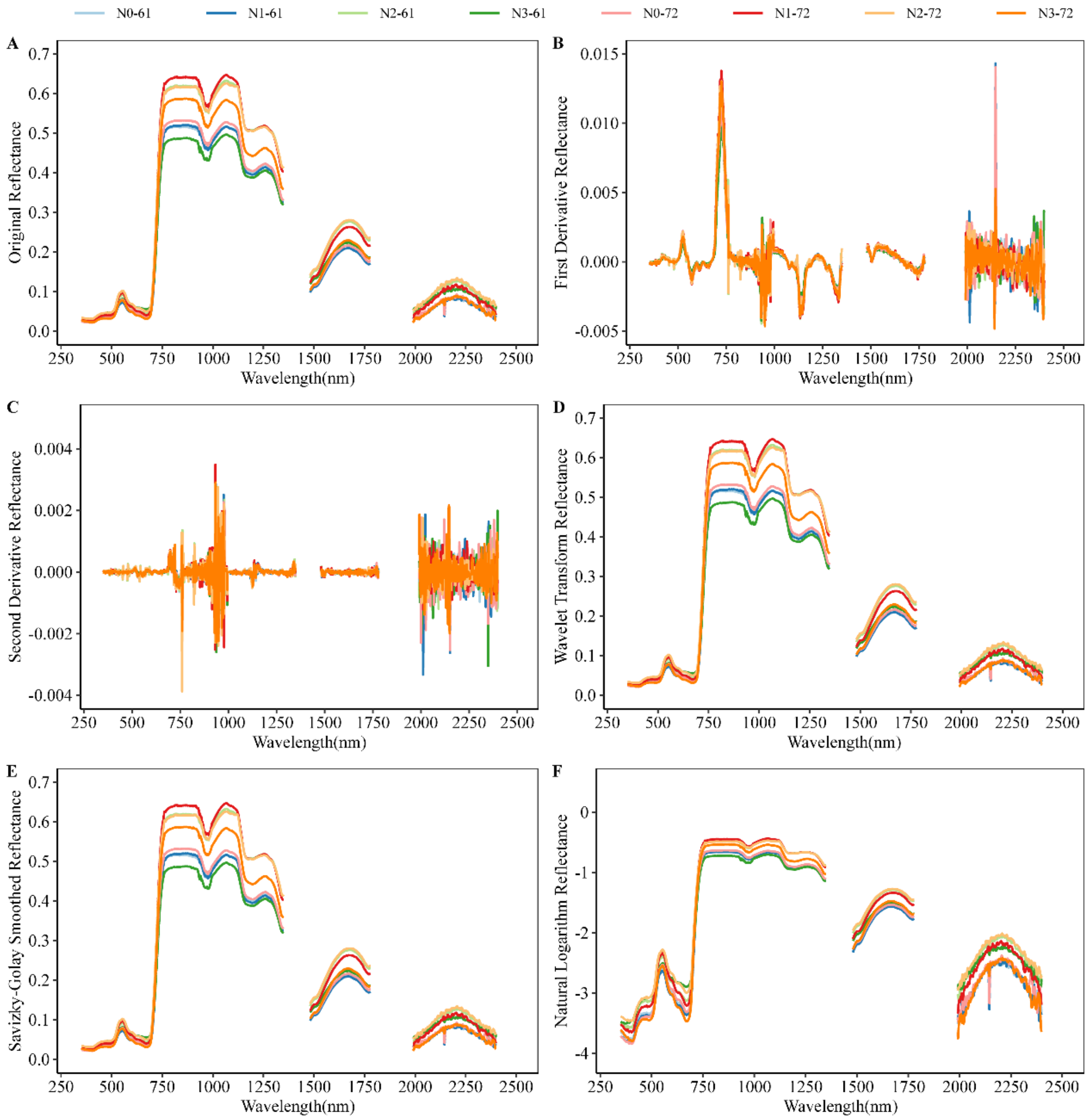

Figure S2. Original and preprocessed spectral data of two cotton varieties under different nitrogen treatments at full flowering stage in Test2019. Notes: The order of pictures is the same as in Figure 3.

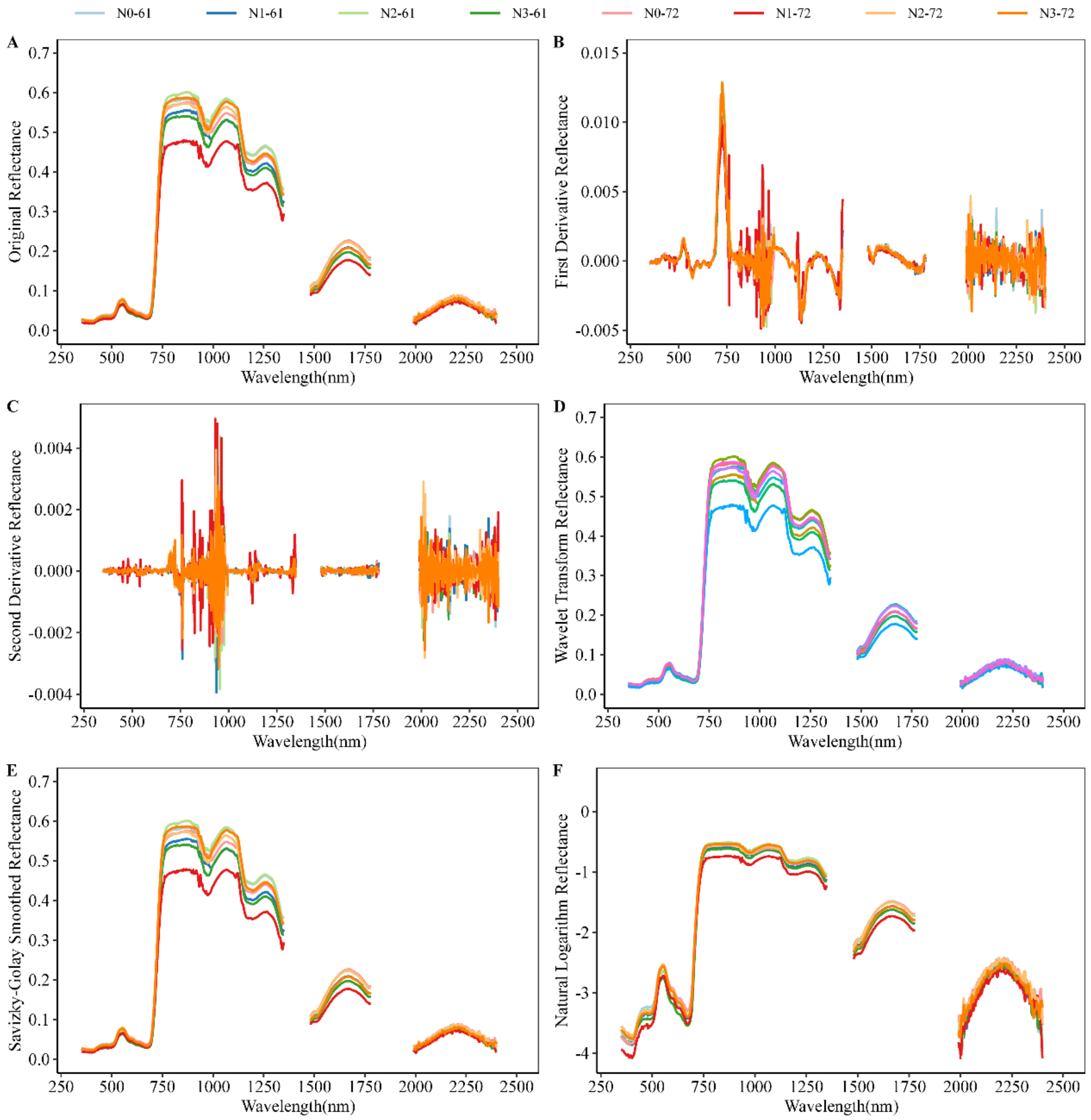

Figure S3. Original and preprocessed spectral data of two cotton varieties under different nitrogen treatments at flower and boll stage in Test2019. Notes: The order of pictures is the same as in Figure 3.

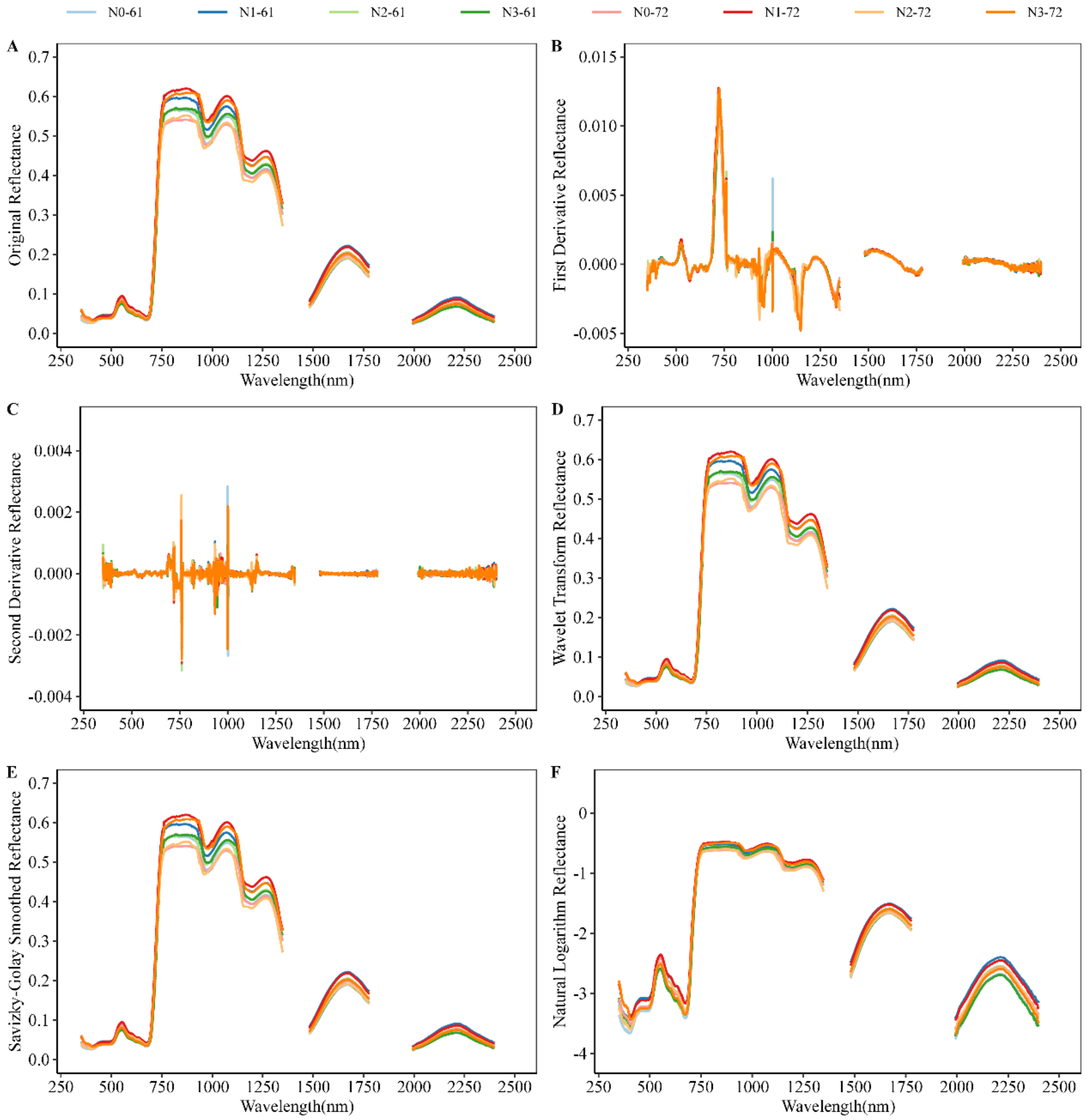

Figure S4. Original and preprocessed spectral data of two cotton varieties under different nitrogen treatments at full boll stage e in Test2019. Notes: The order of pictures is the same as in Figure 3.

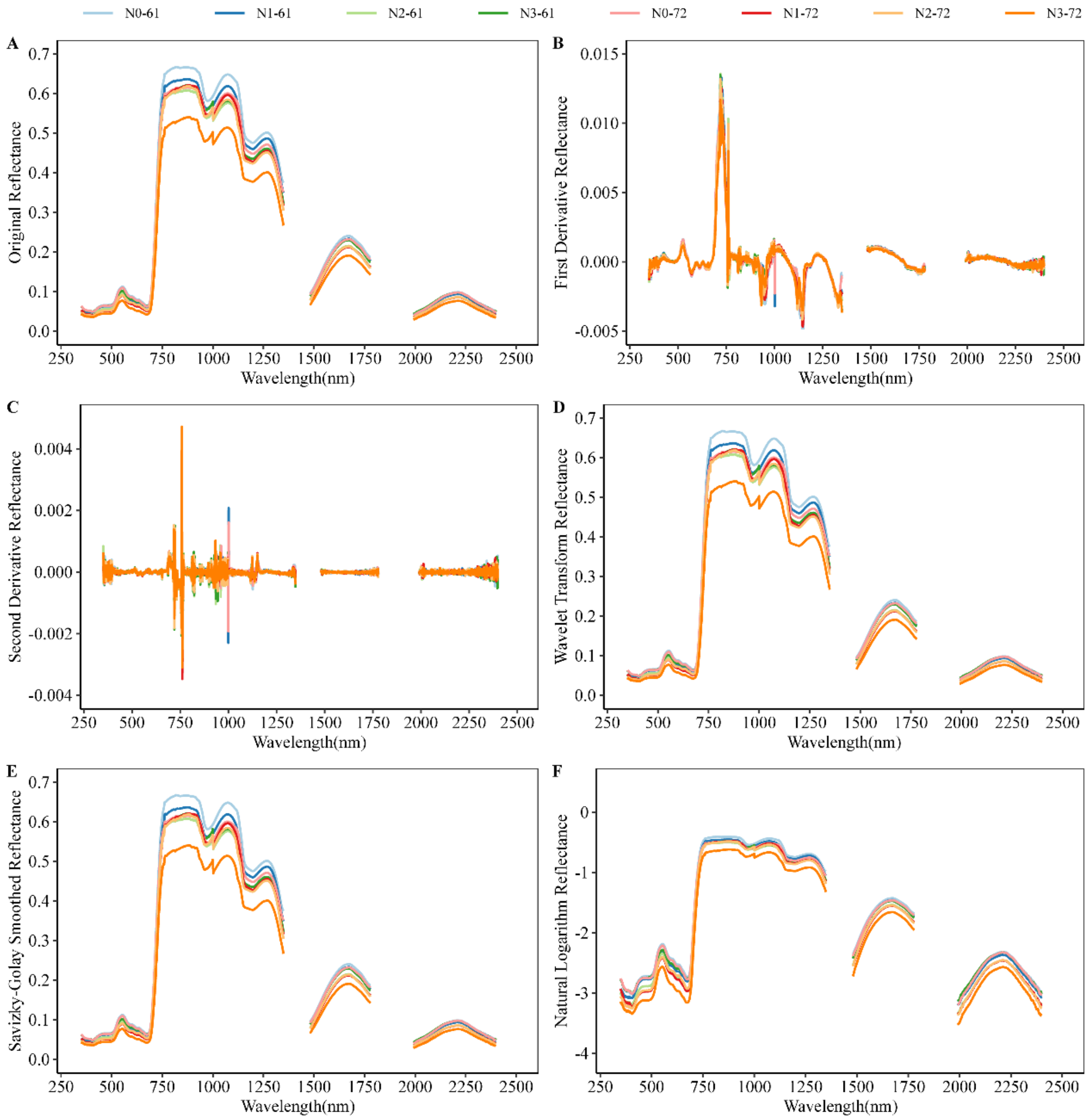

Figure S5. Original and preprocessed spectral data of two cotton varieties under different nitrogen treatments at early boll opening stage in Test2019. Notes: The order of pictures is the same as in Figure 3.

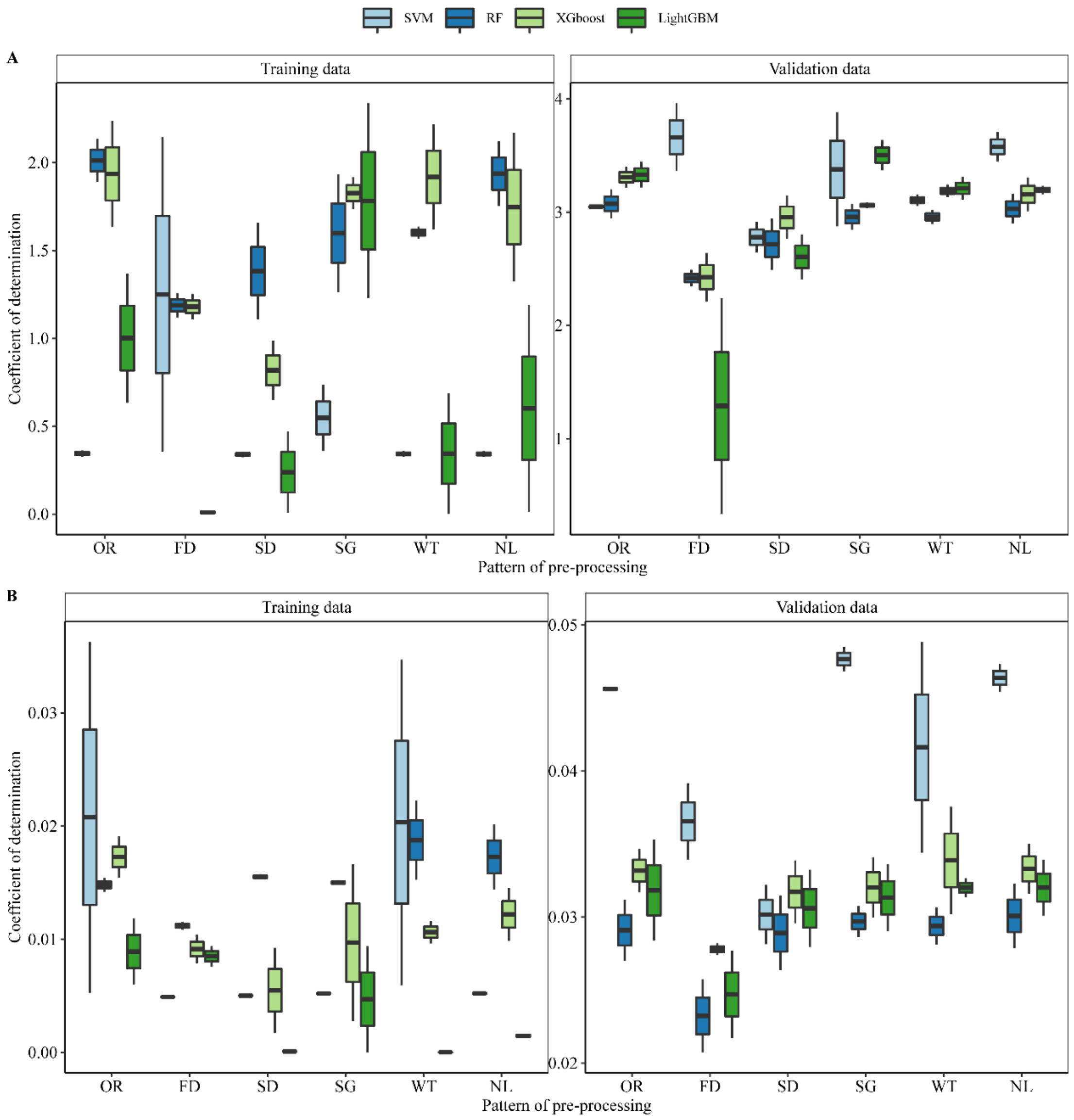

Figure S6. The coefficient of determination (R^2) for each regression model and pre-processing of reflectance. Notes: A: The machine learning models for Pn. B: The machine learning models for FAPAR.

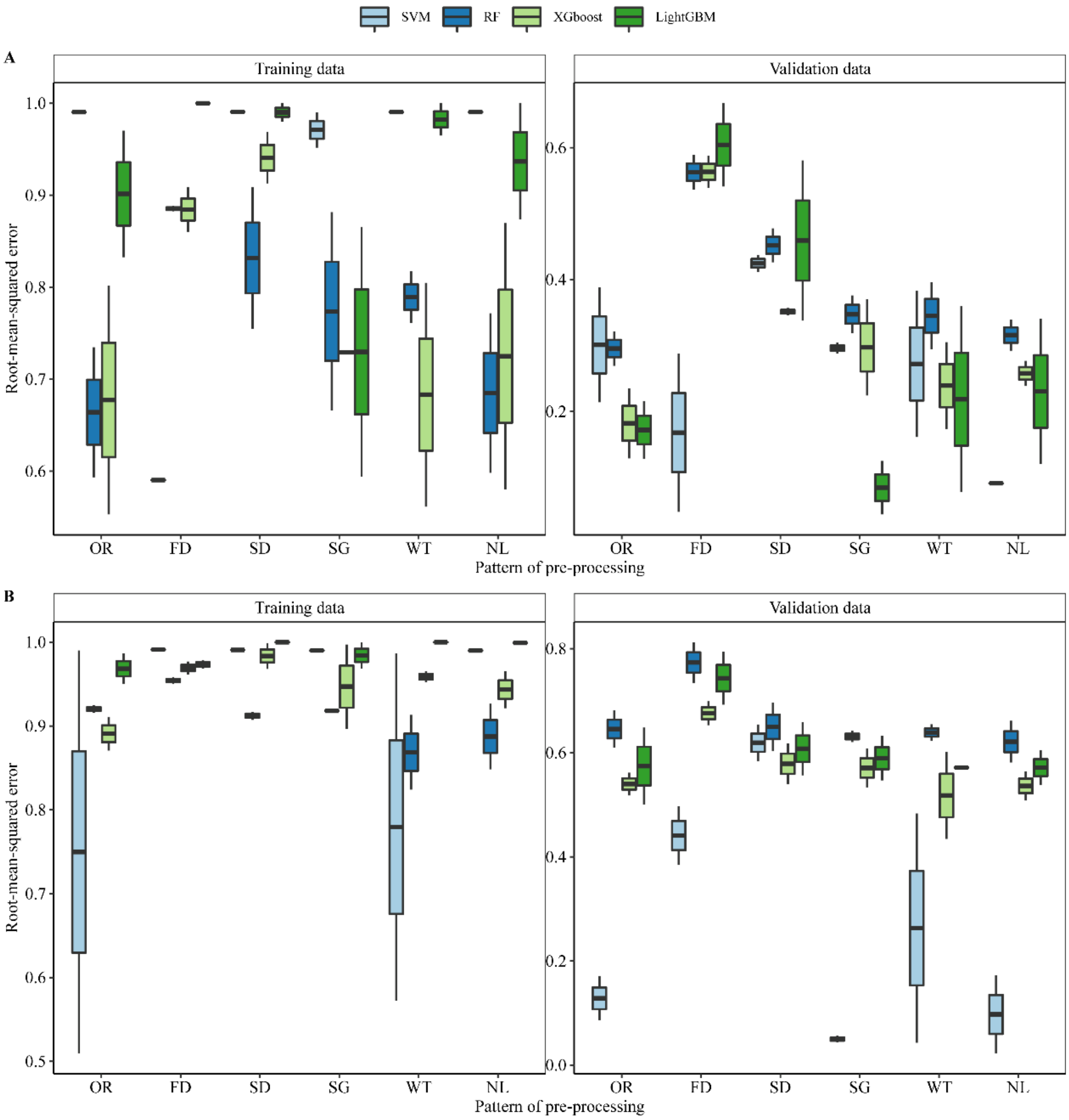

Figure S7. The root mean squared error (RMSE) for each regression model and pre-processing of reflectance. Notes: A: The machine learning models for Pn. B: The machine learning models for FAPAR.

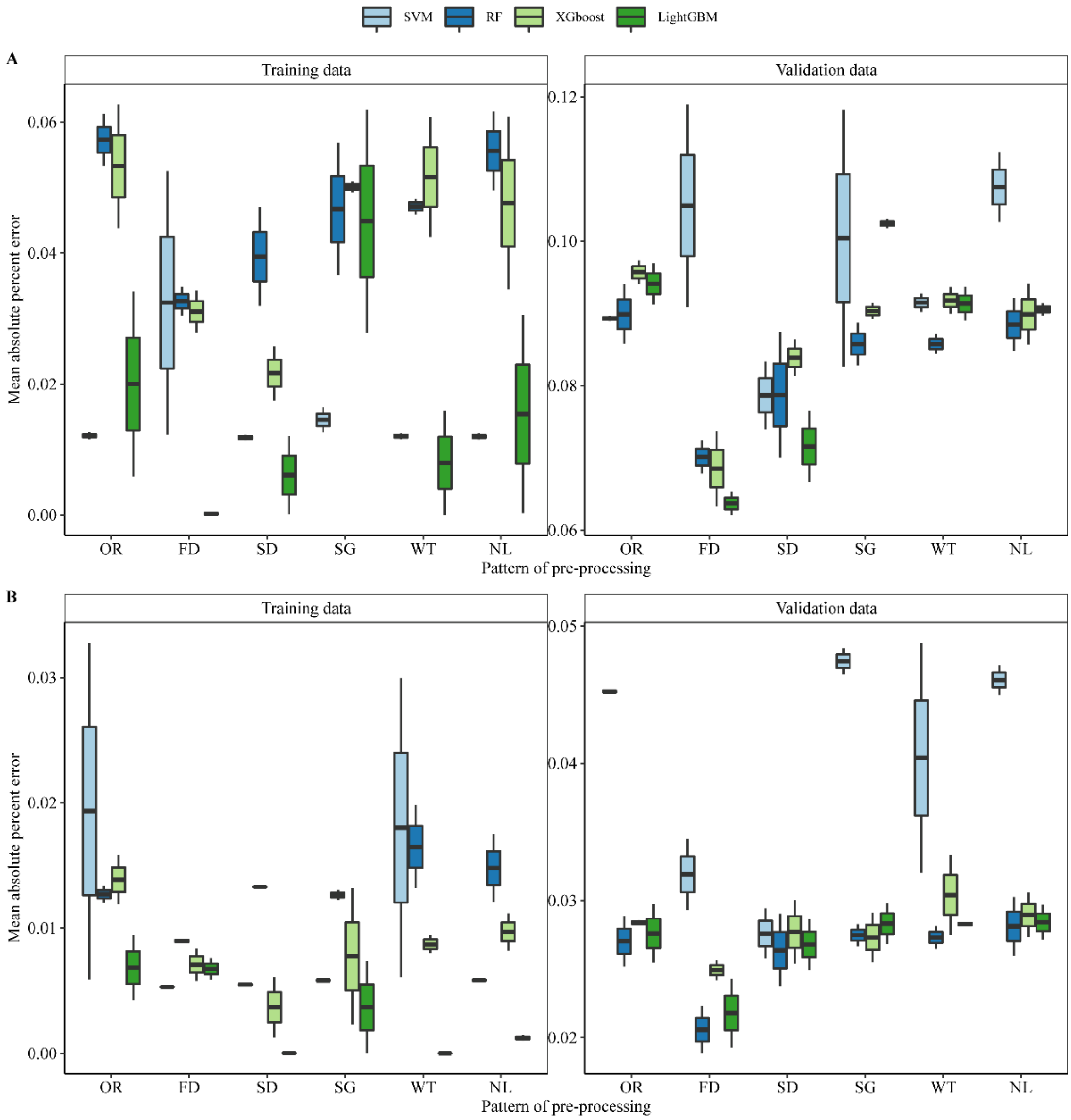

Figure S8. The mean absolute percent error (MAPE)for each regression model and pre-processing of reflectance. Notes: A: The machine learning models for Pn. B: The machine learning models for FAPAR.

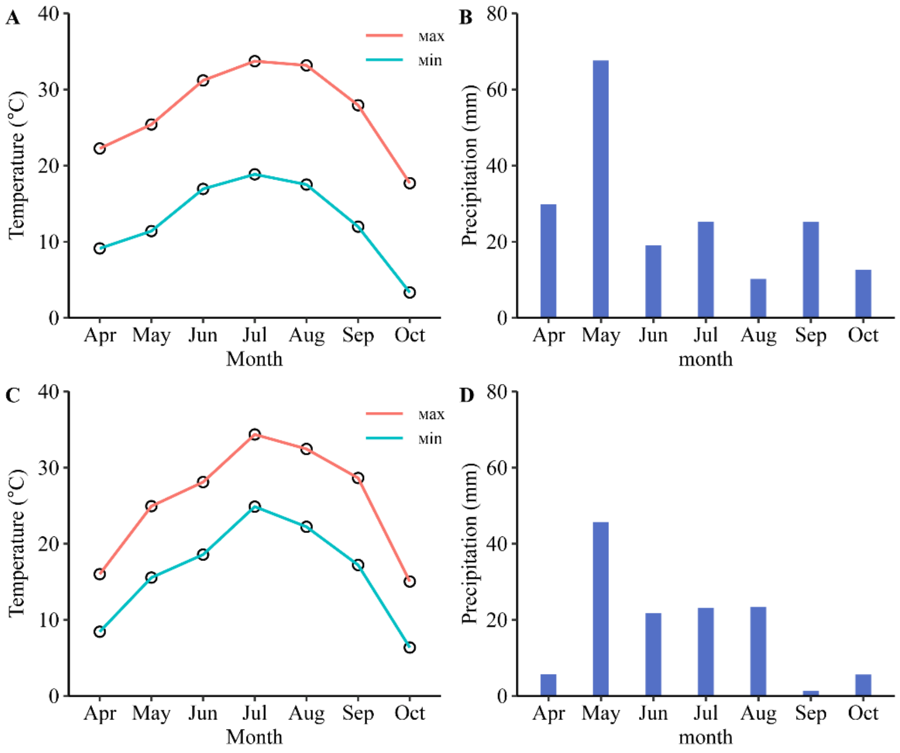

Figure S9. The temperature and precipitation changes of Shihezi, Xinjiang in 2019 and 2021 from sowing to harvesting of cotton. Notes: Weather data from April to October of 2019(A, B) and 2021(C, D) were collected from https://www.worldweatheronline.com.
